# Protocol for sorting cells from mouse brains labeled with mosaic analysis with double markers by flow cytometry

**DOI:** 10.1016/j.xpro.2023.102771

**Published:** 2023-12-08

**Authors:** Nicole Amberg, Giselle Cheung, Simon Hippenmeyer

**Affiliations:** 1 Institute of Science and Technology Austria (ISTA), Am Campus 1, 3400 Klosterneuburg, Austria

**Keywords:** Single Cell, Flow Cytometry, Developmental biology

## Abstract

Mosaic analysis with double markers (MADM) technology enables the generation of genetic mosaic tissue in mice and high-resolution phenotyping at the individual cell level. Here, we present a protocol for isolating MADM-labeled cells with high yield for downstream molecular analyses using fluorescence-activated cell sorting (FACS). We describe steps for generating MADM-labeled mice, perfusion, single-cell suspension, and debris removal. We then detail procedures for cell sorting by FACS and downstream analysis. This protocol is suitable for embryonic to adult mice.

For complete details on the use and execution of this protocol, please refer to Contreras et al. (2021).^1^

## Highlights

• Single-cell suspension from mosaic analysis with double markers (MADM) mouse brains
• Step-by-step guidance through gradient-based debris removal before sorting
• Detailed explanation of parameters and settings for sorting at distinct cytometers
• Outline of downstream molecular analyses and required sorting modalities

## Abstract (editor highlights)

Publisher’s note: Undertaking any experimental protocol requires adherence to local institutional guidelines for laboratory safety and ethics.

## Graphical abstract

**Figure:**
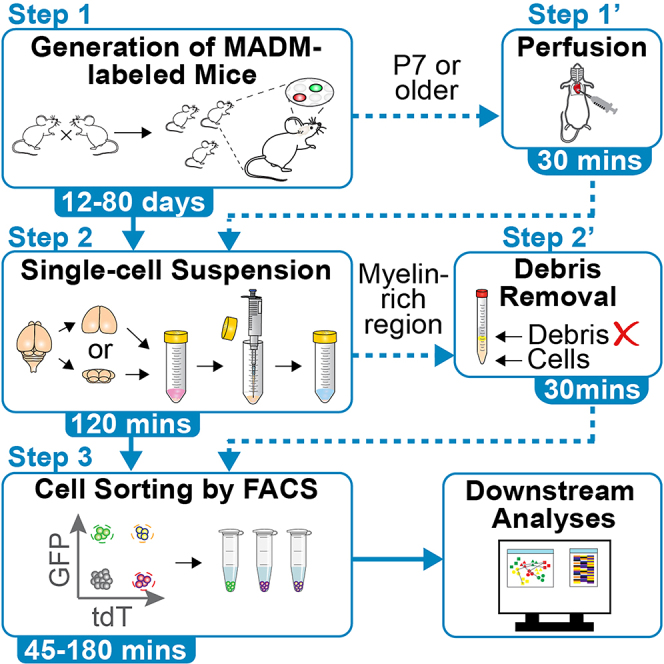

## Before you begin

### Background

This protocol illustrates the sorting of fluorescently labeled cells from genetic mosaic mice using Mosaic Analysis with Double Markers (MADM) and flow cytometry.^1^^,^^2^

A genetic mosaic individual consists of cells with distinct genotypes, for example harboring homozygous mutant cells next to wild-type cells. This phenomenon occurs naturally and is widespread across multicellular organisms. MADM technology allows for a controlled sparse induction of genetic mutations with concomitant fluorescent labeling in experimental animals. As a result, individual wild-type and mutant cells can be unambiguously identified based on distinct fluorescent reporter expression. Thus, MADM mosaicism enables the study of cell-autonomous gene function at single-cell resolution. MADM-labeled cells can therefore be studied at high spatiotemporal resolution in many ways, for example in lineage tracing experiments, cell competition assays, morphological analysis, disease modeling, and can also be subjected to molecular analyses such as transcriptomic or proteomic studies.^1^^,^^3^^,^^4^^,^^2^^,^^5^^,^^6^^,^^7^^,^^8^^,^^9^^,^^10^^,^^11^ Importantly, in any given MADM paradigm the existence of sparse green (GFP^+^) mutant, red (tdT^+^) wild-type and yellow (GFP^+^/tdT^+^) heterozygous cells in an otherwise unlabeled heterozygous environment not only allows us to study gene function, but also to perform gene dosage analysis at the individual cell level within the same tissue, in a cell-type-specific manner.^1^^,^^11^ To achieve these goals, it is crucial to isolate and collect distinct populations of MADM-labeled cells with high yield, quality and purity. However, the dual-color nature and sparseness of MADM-labeling may present some technical challenges.

**Figure 1:**
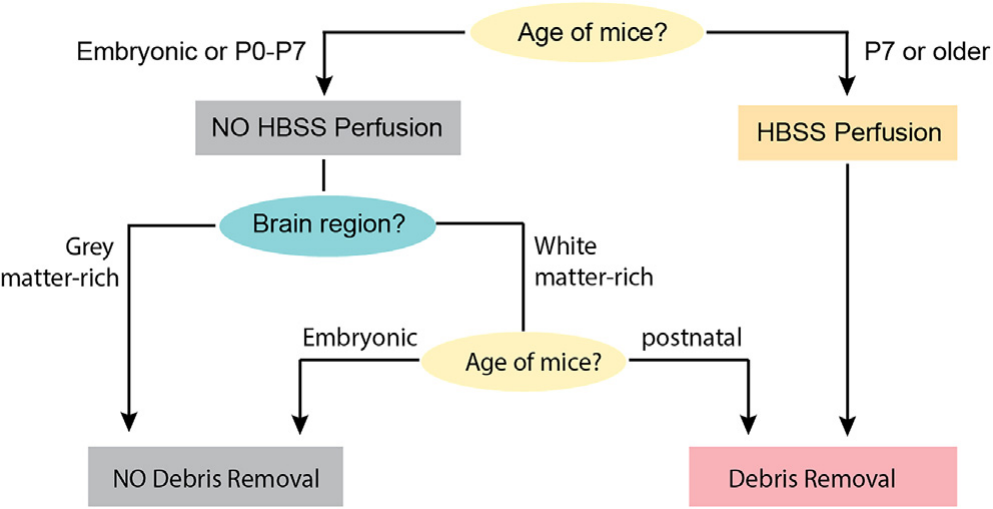
Flow chart summarizing the workflow and considerations for the preparation of single-cell suspensions from MADM-labeled brains

In this protocol, we present an experimental pipeline for the isolation of fluorescently labeled cell populations from MADM-labeled mosaic brains by flow cytometry and highlight a variety of downstream analysis applications. Our protocol is based on the use of neural stem cell-specific Cre drivers in combination with MADM cassettes targeted to all 19 mouse autosomes.^1^^,^^12^ We illustrate distinct optimized protocols for the generation of single-cell suspensions from embryonic and postnatal brain tissue from genetic mosaic MADM mice. Given the sparse nature of MADM labeling, these protocols take into account low cell yield and are optimized for high cell survival and efficient sample collection. A number of considerations are summarized as a flow chart in Figure 1.

For more details on the MADM technology, please refer to our earlier studies.^1^^,^^2^^,^^9^ For the successful generation of experimental MADM mice, please follow the instructions provided in our published experimental protocol.^12^

***Note:*** For MADM to work, the presence of a Cre recombinase, active in stem or progenitor cells of your tissue of interest, is required. In other words, any Cre-driver can be used, provided Cre is expressed in dividing stem and progenitor cells, to target cell types and lineages of interest. If working with a new Cre driver, we recommend to first test and validate promoter activity in stem/progenitor cells of interest, e.g., by confirming transcript or protein levels of the respective gene, in publicly available datasets. Particularly single-cell transcriptome datasets offer an excellent tool to assess stem/progenitor cell specific expression of the promoter of interest. As a next step, we suggest to perform *in vivo* validation of Cre activity by crossing the Cre driver line with a reporter model harboring a lox-STOP-lox cassette upstream of a fluorescent reporter sequence. Analysis verifying reporter expression in stem and progenitor cells by histological co-staining for cell-type specific markers should be performed at time points suitable for the tissue of interest. Once Cre activity in the stem/progenitor population of interested is confirmed, a MADM experiment can be conducted. Readers are recommended to refer to a detailed protocol^12^ for the generation of MADM mice and breeding schemes.

### Institutional permissions

Institutional and governmental permission and oversight information for the animal study should be obtained. In this study, experimental procedures were discussed and approved by the institutional ethics and animal welfare committees at IST Austria in accordance with good scientific practice guidelines and national legislation (license number: BMWF-66.018/0007-II/3b/2012 and BMWFW-66.018/0006-WF/V/3b/2017).

## Key resources table

**Table:** 

REAGENT or RESOURCE	SOURCE	IDENTIFIER
**Chemicals, peptides, and recombinant proteins**		

Papain vial	Worthington Biochemical	Cat#PAP2
DNase vial	Worthington Biochemical	Cat#D2
Ovomucoid inhibitor vial	Worthington Biochemical	Cat#OI-BSA
EBSS	Thermo Fisher Scientific	Cat#24010043
DMEM/F12	Thermo Fisher Scientific	Cat#21041025
Heat-inactivated fetal bovine serum (FBS)	Thermo Fisher Scientific	Cat#10082147
Heat-inactivated horse serum (HS)	Thermo Fisher Scientific	Cat#26050088
Hanks’ balanced salt solution - HBSS (1X) -CaCl_2_, -MgCl_2_	Thermo Fisher Scientific	Cat#14175095
Dulbecco’s phosphate-buffered saline - DPBS (1X) -CaCl_2_, -MgCl_2_	Thermo Fisher Scientific	Cat#14190094
Debris removal solution	Miltenyi Biotec	Cat#130109398
D-(+)-trehalose dehydrate	Merck	Cat#90210
D-(-)-2-amino-5-phosphonopentanoic acid (APV)	Merck	Cat#A8054
Avertin (2,2,2 tribromoethanol)	Sigma-Aldrich	Cat#T48402
t-amylalcohol (2-methyl-2-butanol)	Sigma-Aldrich	Cat#240486
Triton X-100	Sigma-Aldrich	Cat#T878
RNase inhibitor	Takara	Cat#2313A
RNase AWAY	Thermo Fisher Scientific	Cat#10328011
RNase-free water	Thermo Fisher Scientific	Cat#10977035

**Critical commercial assays**		

Chromium Next GEM Single Cell 3′ Reagent Kits v3.1	10× Genomics	Cat#PN-1000121
iST-NHS Kit	PreOmics	Cat#P.O.00026

**Experimental models: Organisms/strains**		

*Mus musculus*: MADM-19-GT (2 months–1 year old; both sexes)	Contreras et al.^1^ European Mouse Mutant Archive (EMMA)	RRID:IMSR_EM:14720
*Mus musculus*: MADM-19-TG (2 months–1 year old; both sexes)	Contreras et al.^1^ EMMA	RRID:IMSR_EM:14721
*Mus musculus*: *Nestin*-Cre (2 months–1 year old; both sexes)	Petersen et al.^13^	N/A

**Software and algorithms**		

FACSDiva	BD Biosciences	https://www.bdbiosciences.com/en-ca/products/instruments/software-informatics/instrument-software/bd-facsdiva-software-v-6-1-3.643629
Sony SH800 software	Sony	https://www.sonybiotechnology.com/us/instruments/sh800s-cell-sorter/software/

**Other**		

Microfuge tubes 1.5 mL	Sarstedt	Cat#62.547.254
50 mL centrifuge tubes	Nalgene	Cat#194-2520
15 mL centrifuge tubes	Sarstedt	Cat#62.554.502
0.2 micron filter	Nalgene	Cat#194-2520
Dissection microscope	Zeiss Stemi DV4	N/A
Petri dish	Thermo Scientific	Cat#NC9565080
60 mL syringe	BD Biosciences	Cat#300185
20 mL syringe Omnifix	Braun	Cat#4617207V
Needle 20G Sterican	Braun	Cat#4657705
Centrifuge Heraeus Multifuge X3R	Thermo Scientific	Cat#15334336
FACSAria III	BD Biosciences	N/A
Sony SH800	Sony	N/A
AluminaSeal	Merck	Cat#Z740251-100EA
Hard-Shell PCR plates (96-well)	Bio-Rad	Cat#HSP9631
PCR tubes & caps, RNase-free, 0.2 mL (8-strip format)	Thermo Fisher Scientific	Cat#AM12230
Falcon 5 mL polystyrene round-bottom tube with cell strainer cap (Falcon 352235) (40 μm cell strainer)	Fisher Scientific	Cat#10585801
Microfuge tubes 1.5 mL	Thermo Fisher Scientific	Cat#AM12450
TPP TubeSpin 50 mL bioreactor tubes	Merck	Cat#Z761028-180EA
Dissection tools	FST	Various, depending on the specific needs of the experiment
Shaking water bath SBS40	Cole-Parmer Stuart	N/A

## Materials and equipment

### Essential buffers on the day of the experiment

**Timing: 1 h**

### Solutions from Worthington papain kit

Resuspend Papain containing L-cysteine & EDTA (in 5 mL EBSS), DNase (in 0.5 mL EBSS) and Ovomucoid protease inhibitor (in 32 mL EBSS) according to manufacturer’s instructions.

Papain and DNase should be prepared freshly on the day of the experiment.

Resuspended Ovomucoid protease inhibitor can be stored at 4°C and used for several months.

**Table:** Papain solution

Reagent	Final Concentration	Amount
Papain	20 units/mL	1 vial
EBSS	N/A	5 mL

Keep at 4°C until preparing the Papain-DNase solution.

**Table:** DNase solution

Reagent	Final Concentration	Amount
DNase	1,000 units/mL	1 vial
EBSS	N/A	500 μL

Keep at 4°C until preparing the Papain-DNase solution.

**Table:** Papain-DNase solution

Reagent	Final Concentration	Amount
Papain	20 units/mL	5 mL
DNase	166.7 units/mL	250 μL

Keep at 4°C for maximum 24 h. Pre-activate by incubating at 22°C–25°C for 30 min.

**Table:** Solution 2

Reagent	Final Concentration	Amount
EBSS	N/A	5,350 μL
DNase	166.7 units/mL	250 μL
Ovomucoid protease inhibitor	0.67 mg	400 μL
**Total**	N/A	6,000 μL

Keep at 4°C for maximum 24 h.

### Additional solutions

**Table:** Trehalose (50%)

Reagent	Final concentration	Amount
Trehalose	50%	10 *g*
RNase free H_2_O	N/A	20 mL
**Total**	**50%**	**20 mL**

Details for trehalose preparation:

• Dissolve Trehalose in RNase free H_2_O at 55°C for 30 min, shaking occasionally.
• Store at 22°C–25°C for up to 1 week.

**Table:** APV

Reagent	Final concentration	Amount
APV	25 mM	5 mg
RNase free H_2_O	N/A	1 mL
**Total**	**25 mM**	**1 mL**

Details for APV preparation:

• Dissolve APV in RNase free H_2_O.
• Make aliquots of 100 μL and store at ‒20°C.

**Table:** Anesthesia stock solution (100%)

Reagent	Final concentration	Amount
Avertin	100%	7 *g*
t-amylalcohol	N/A	7 mL
**Total**	**100%**	**7 mL**

Details for anesthesia preparation:

• Vortex until the solution appears homogeneous.
• Wrap in tin foil to protect from light and store at 22°C–25°C for up to 6 months.

**Table:** Anesthesia working solution (2.5%)

Reagent	Final concentration	Amount
100% anesthesia stock solution	2N	0.875 mL
1X PBS	1X	34.125 mL
**Total**	**2.5%**	**35 mL**

Details for anesthesia preparation:

• Vortex until the solution appears homogeneous.
• Filter the solution into a fresh tube using a 60 mL syringe and a 0.2 micron filter.
• Store at 4°C for up to one month.

**Table:** EBSS with cell survival supplements

Reagent	Final Concentration	Amount
EBSS	N/A	17.96 mL
Trehalose (50%)	5%	2 mL
APV (25 mM)	50 μM	40 μL
**Total**	N/A	20 mL

Keep at 4°C for up to 14 days.

**Table:** DMEM/F12 with cell survival supplements

Reagent	Final Concentration	Amount
DMEM/F12	N/A	17.98 mL
Trehalose (50%)	5%	2 mL
APV (25 mM)	25 μM	20 μL
**Total**	N/A	20 mL

Keep at 4°C for up to 14 days.

**Table:** Media

Reagent	Final Concentration	Amount
DMEM/F12	N/A	14.4 mL
FBS	10%	1,800 μL
HS	10%	1,800 μL
**Total**	N/A	18 mL

Keep at 4°C for up to 14 days.

**Table:** RNA Lysis buffer

Reagent	Final Concentration	Amount
Triton X-100	0,2%	0.08 μL
RNase Inhibitor	2 units/μL	0.2 μL
RNase free water	N/A	3.72 μL
**Total/well**	N/A	4 μL

Keep at 4°C and always prepare freshly on the day of the experiment.

***Note:*** This lysis buffer can be used to collect single cells for scRNA-seq or low inputs of bulk (up to 400 cells). In our lab, RNA-seq with these amounts of cells was successfully performed using the Smart-seq V2 protocol.^14^

## Step-by-step method details

### Generation of experimental MADM mice for FACS isolation of neuronal cells with MADM-induced mosaicism

**Timing: 12–80 days**

This section of the protocol describes the generation of experimental MADM mice.

1. Cross *MADM*^*TG/TG,gene-X*^ animals with *MADM*^*GT/GT*^*;Nestin-*Cre animals. In order to generate *MADM*^*TG/TG,gene-X*^ animals and *MADM*^*GT/GT*^*;Nestin-*Cre animals please refer to the step-by-step protocol provided by our group^12^ (see Troubleshooting Problem 1).
  a. Check for vaginal plug to monitor successful mating and to facilitate the experimental planning (see Troubleshooting Problem 2).
  b. Males and females of the F1 generation can be used as experimental animals (genotype is either control [*MADM*^*GT/TG*^*;Nestin-*Cre] or genetic mosaic [*MADM*^*GT/TG,gene-X*^*;Nestin-*Cre]).
  c. Perform experiment at developmental stage(s) of choice.

***Note:*** Experimental animals can be used at embryonic stages, P0, early postnatal stages and up to adulthood (>P21). Here we made use of *Nestin*-Cre that is expressed in neural stem cells in both the central nervous system and the peripheral nervous system.^13^ Based on our experience, the protocol will most efficiently work with embryonic up to P0 brains, which show a low percentage of debris and exhibit high cell survival rate. In general, the time required for sorting increased with the age of the experimental animal due to an increase of myelin-containing debris in the single-cell suspension.

***Note:*** Here we describe the usage of *Nestin-*Cre. Besides *Nestin-*Cre*,* any other *Cre*-driver can be used, provided Cre is expressed in dividing stem and progenitor cells, to target cell types and lineages of interest. Depending on the use of specific chromosome with targeted MADM cassettes and the particular Cre driver, interchromosomal recombination and MADM-labeling efficiencies in experimental animals might vary^1^ (see Troubleshooting Problem 3).

***Note:*** If your experimental needs require sample pooling, perform genotyping with your standard genotyping protocol for your experimental mice to make sure your sample pool consists of the correct genotypes before you proceed with the protocol.

### Preparation of single-cell suspension from MADM-labeled mouse brains

**Timing: 1.5–3 h**

This section of the protocol documents the dissection and preparation of single-cell suspension from MADM-labeled mouse brains for sorting of fluorescent cells.

### Prepare equipment (on the day of experiment) and required solutions:

**Timing: 30 min**

2. Clean all dissection tools (2 curved forceps and scissors) with 70% ethanol and RNase Away, a decontamination reagent that removes RNase from equipment and surfaces.
3. Pre-warm shaking water bath (37°C) for single-cell suspension of desired tissue.
4. Prepare collection tubes and respective buffer to sort your cells in.
  ***Note:*** The type of tube depends on your downstream analysis: use 1.5 mL microfuge tubes for 10× genomics single-cell RNA-seq experiments, 1.5 mL microfuge tubes for proteomics, or 0.2 mL PCR tubes for bulk RNA-seq with SMART-seq technology.
  ***Note:*** Since neither sorting device can hold 0.2 mL PCR tubes in its collection tube holders, prepare custom-made PCR tube holders for sorting:
  a. Take 1.5 mL microfuge tubes and cut off the lid.
  b. Fill the 1.5 mL tubes with paper tissue or Parafilm (Figure 6C).
  c. Insert a PCR tube in the middle of the stuffed 1.5 mL tube.
  d. After sorting, PCR tubes can be taken out of the 1.5 mL tubes with forceps and PCR tube holders can be reused by inserting a new PCR tube.
    ***Note:*** Here, we describe dissociation of the mouse cortex and midbrain as two examples of gray and white matter-rich tissues, respectively. However, this protocol can be used for all regions of the brain with suitable adaptations.
    ***Note:*** For more efficient dissociation and yield, mice P7 or older should first be perfused transcardially with cold HBSS before tissue isolation to remove blood from the brain. Additional debris removal steps (steps 30–38) based on a density-gradient are recommended when isolating white matter-rich regions like the cortex from mice ≥ P7, or the midbrain from mice ≥ P0 (Figure 1). Debris not only includes dead cells, but also myelin fragments. With increasing levels of myelination in distinct brain regions, the amount of debris in your single-cell suspension increases. Thus, debris removal will help to increase the yield of your viable cell population for your FACS experiment.
    **CRITICAL:** For brains requiring perfusion, supplement EBSS and DMEM/F12 with cell survival supplements in subsequent dissociation steps.
    **CRITICAL:** There is no stopping point in the protocol after starting with dissection of the experimental animal until the end of sorting.
5. Prepare all solutions listed below required for tissue dissociation:
  a. Cold HBSS (for dissection of embryonic brains and perfusion of postnatal brains only).
  b. EBSS or EBSS with cell survival supplements.
  c. Papain-DNase solution.
  d. Solution 2.
  e. DMEM/F12 or DMEM/F12 with cell survival supplements.
  f. Media.
  g. Cold PBS (for debris removal steps only).
  h. Debris removal solution (for debris removal steps only).

**CRITICAL:** Adjust volume of Papain-DNase solution and Solution 2 according to amount of tissue per enzymatic digestion reaction.

**Table:** Volumes of reagents are adjusted according to the amount of tissue/sample used

Tissue per reaction	Papain-DNase solution	Solution 2 (step 24)	Solution 2 (step 26)
Single embryonic cortex/midbrain	650 μL	500 μL	250 μL
Up to 3 embryonic cortices/10 embryonic midbrains)	1,300 μL	1,200 μL	300 μL
Single postnatal cortex	1,300 μL	1,200 μL	300 μL
Up to 3 postnatal cortices/ 5 postnatal midbrains)	2,600 μL	2,400 μL	600 μL

### Dissect brains

**Timing: 10–30 min**

**Figure 2:**
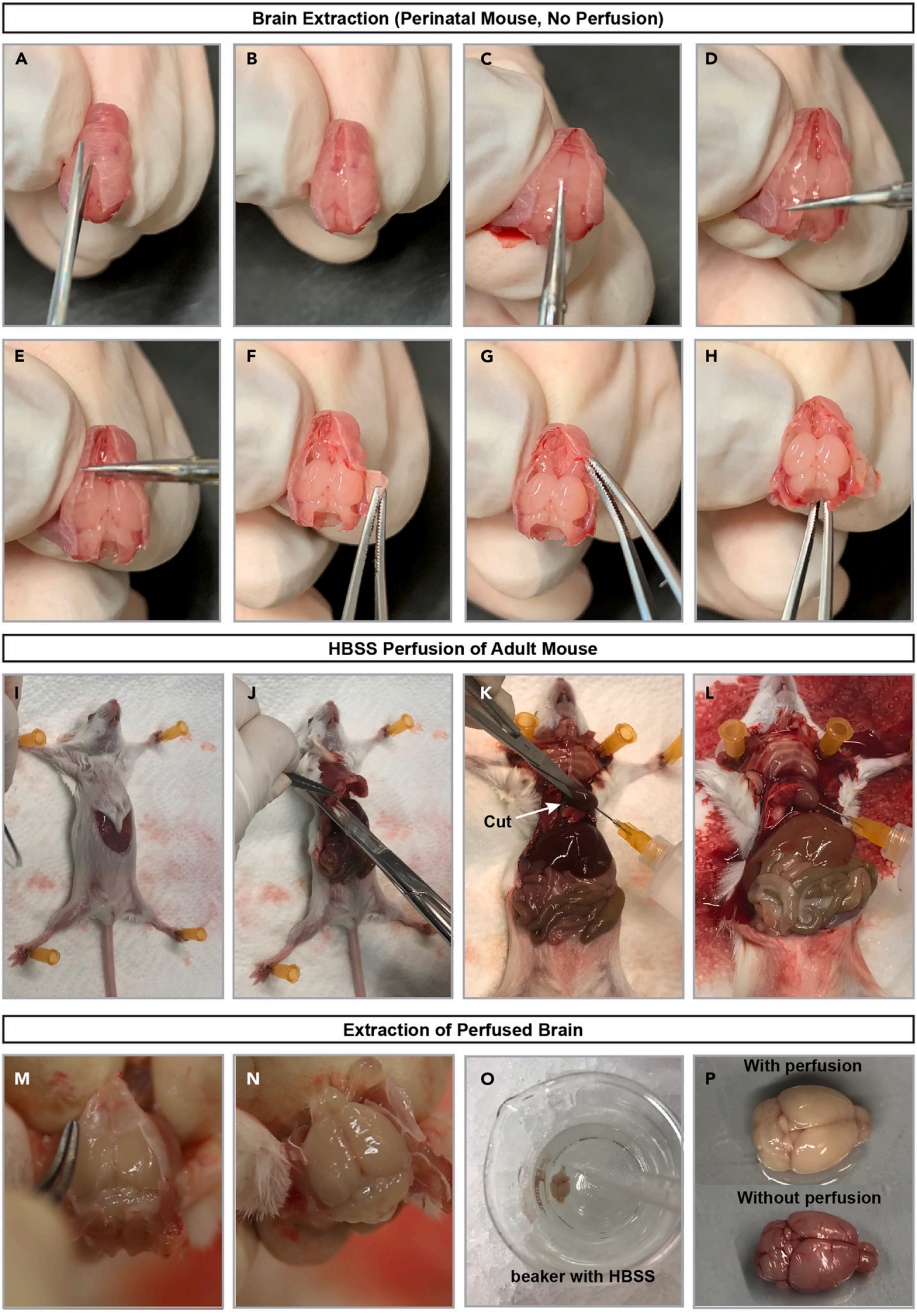
Extraction of MADM-labeled brains (A‒H) Images of stepwise brain extraction from a perinatal mouse. (I‒L) HBSS perfusion of an adult mouse. (M‒O) Images of the stepwise brain extraction from adult, perfused mouse. (P) Comparison of adult brains with (top) and without (bottom) transcardial perfusion.
8. For postnatal mice P7 or older, perform transcardial perfusion prior to dissection to remove blood from the brains (Figures 2I‒2P, detailed in steps 9–20) (see Troubleshooting Problem 4).

***Note:*** This procedure is essential for cell survival and the enrichment of neural cell types in mice with developed brain vasculature systems.

6. For embryos and up to P7 postnatal mice, sacrifice mice by decapitation.
7. Extract brain and dissect brain region of interest of embryos up to E16 in cold HBSS solution in a Petri-dish under a dissection microscope. Between E16 and P7, extract the brains as follows (Figures 2A–2H) and then skip to step 21:
  a. Remove the skin over the skull.
  b. Make a long cut on the top of the skull along the midline.
  c. Make a short cut at the naso-frontal suture (in front of the olfactory bulbs) and two short cuts along the parietal-interparietal sutures.
  d. Flip the cut skull to the side to have access to the brain.
  e. Place curved forceps underneath the brain and remove it from the skull.

9. Anesthetize mice by intraperitoneal injection of anesthesia working solution at a volume of 0.16–0.24 mL/10 *g* mouse (equivalent to 400–600 mg/kg).

**CRITICAL:** If more than one mouse is used, anesthetize mice one at a time immediately prior to perfusion.

**CRITICAL:** It is essential to always ensure that mice are fully anesthetized before continuing with the procedures. This can be done by observing a complete lack of response after pinching their paws with forceps. If mice are still responsive after 5 min of administering anesthesia, more anesthesia working solution can be added in small amounts.

10. Place the anesthetized mouse in supine position on a perfusion tray and secure each foot with a separate needle.
11. Fill a 20 mL syringe with 20 mL of cold HBSS solution and attach a needle. Remove air bubbles and keep on ice until perfusion.
12. Disinfect fur with 70% ethanol.
13. Make a medial incision below the rib cage with scissors and surgical forceps through the skin and the underlying muscle layer. Continue to cut laterally until rib cage is exposed (Figure 2I).
14. Lift the tip of the sternum with forceps and cut along the base of the diaphragm. Cut each sides of the rib cage to expose the heart (Figure 2J).
15. Make an incision to the posterior end of the right atrium using small iris scissors to allow blood to drain during the perfusion (Figure 2K).
16. Insert the needle attached to the 20 mL syringe needle containing cold HBSS into the lower left ventricle of the exposed heart.

**CRITICAL:** Be careful to not puncture the wall into the left atrium (Figure 2K).

17. Press the syringe plunger at a steady pace (up to 20 mL/min depending on the age of the mouse) and perfuse with HBSS until the liver turns pale (Figure 2L).
18. When perfusion is complete, withdraw needle and decapitate mouse.
19. Extract the brains as follows (Figures 2M and 2N):
  a. Remove the skin and skull over the brain.
  b. Make a long cut on the top of the skull along the midline.
  c. Flip the cut skull to the side to have access to the brain.
  d. Place curved forceps underneath the brain and remove it from the skull.
20. Allow perfused brains to rest for 2 min in a beaker containing cold HBSS solution on ice before proceeding (Figure 2O).

**CRITICAL:** A pale color of the brain is an indication of a successful perfusion (Figure 2P).

### Prepare single-cell suspension

**Timing: 45 min to 2 h 15 min**

**Figure 3:**
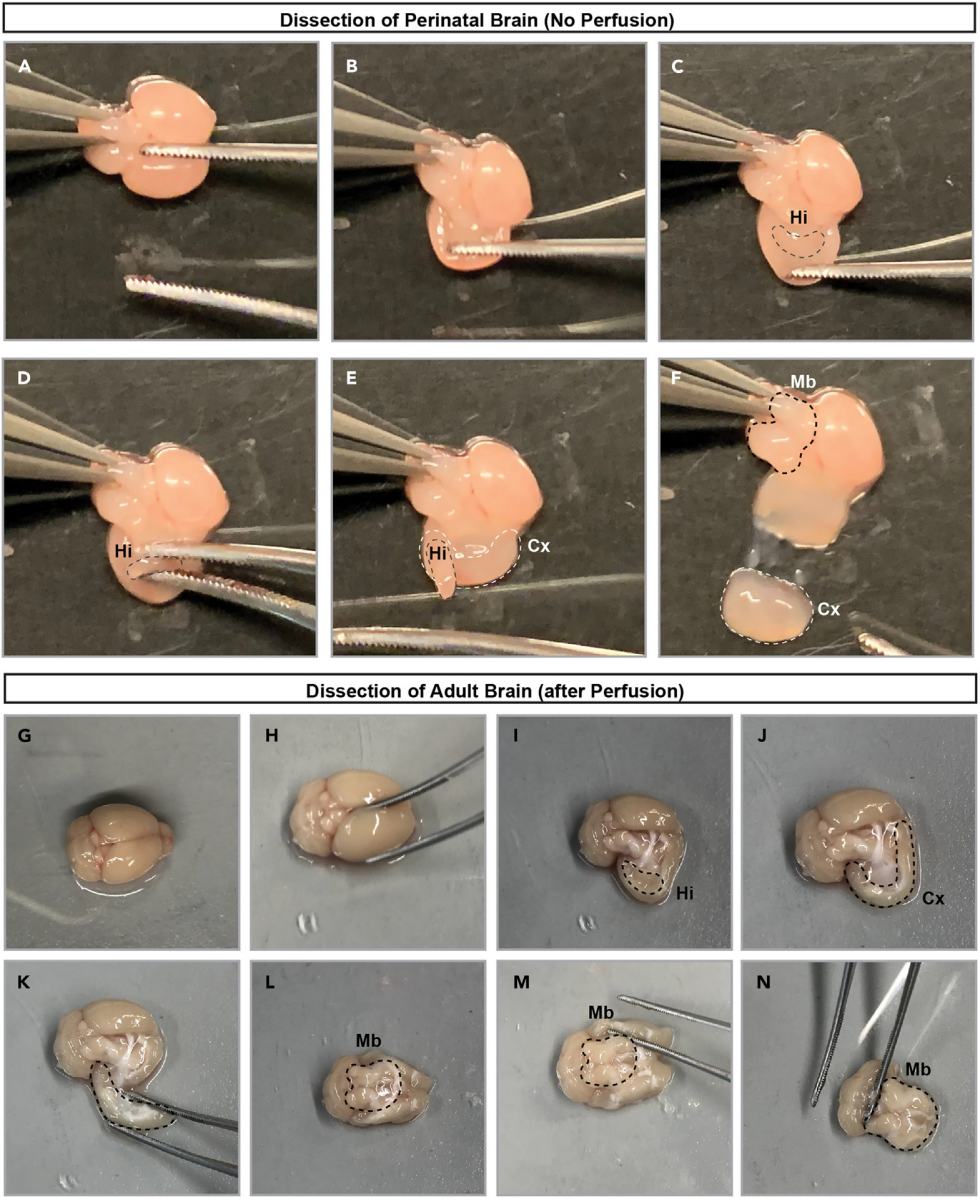
Dissection of MADM-labeled brains (A‒F) Dissection of brain extracted from a perinatal mouse. Dashed lines indicate hippocampus, cortex, and midbrain, respectively. Abbreviations: Hi – hippocampus; Cx – neocortex; Mb – midbrain. (G‒N) Dissection of brain extracted from an adult mouse after perfusion. Dashed lines indicate hippocampus, cortex and midbrain, respectively. Abbreviations: Hi – hippocampus; Cx – neocortex; Mb – midbrain.

***Note:*** For perfused brains, perform dissection on ice cold surface.

***Note:*** For more efficient enzymatic dissociation, cut large pieces of tissue in smaller pieces using a sharp razor.

21. Dissect region of interest (e.g., hippocampus, cortex or midbrain) from isolated brains by placing the brains in a petri dish and using forceps (Figure 3).

**CRITICAL:** In our experience, careful and precise dissection is most efficient to avoid contamination from cell types originating from other brain areas. The stepwise isolation of cortices, hippocampi and midbrain is illustrated (Figures 3A‒3F for perinatal brain; Figures 3G‒3N for adult brain).

**Figure 4:**
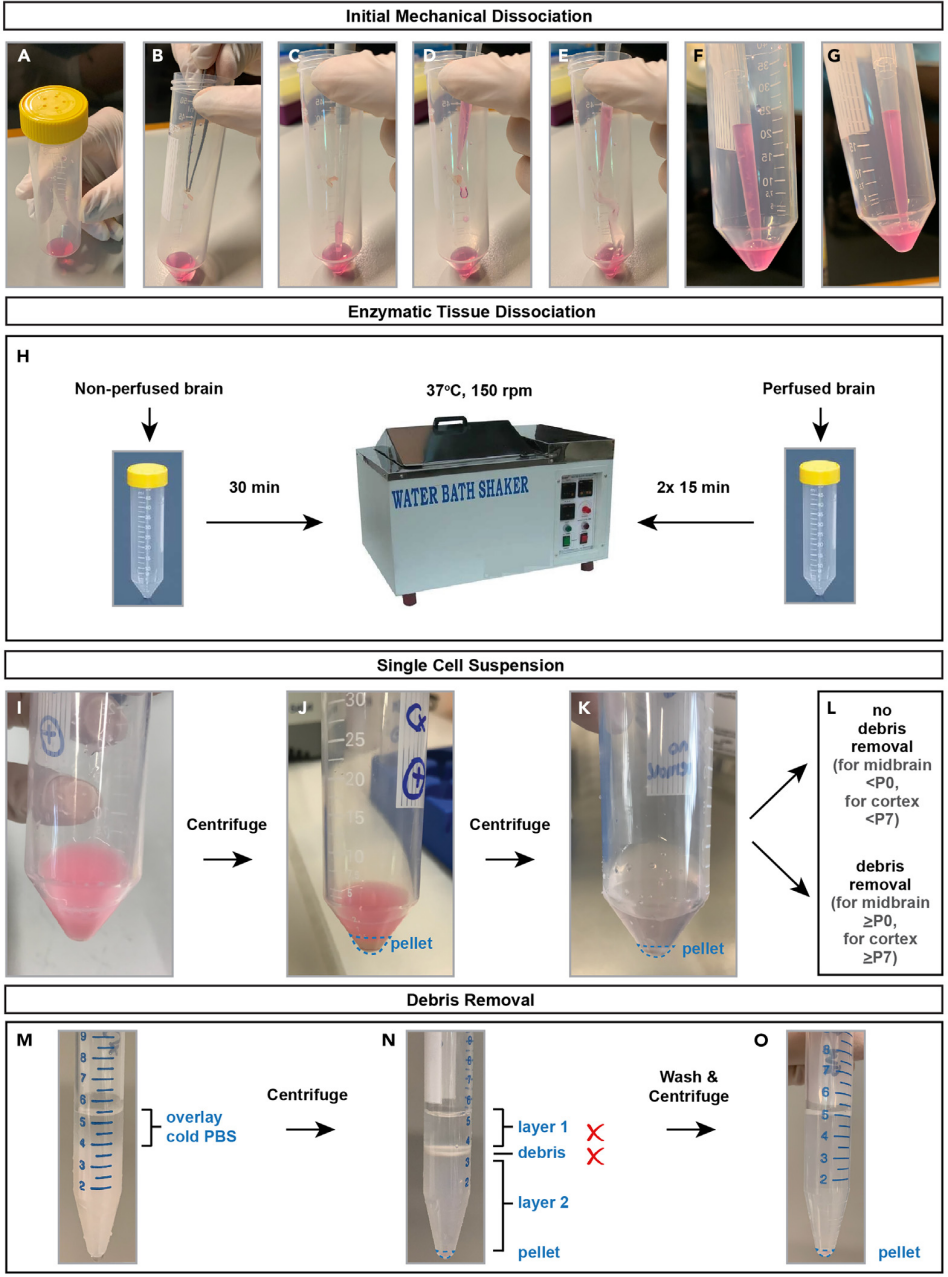
Preparation of single-cell suspensions from MADM-labeled brain regions (A‒G) Transfer of dissected brain region to Papain-DNase solution and initial mechanical dissociation. (H) Workflow of enzymatic tissue dissociation using a shaking water bath. (I‒L) Wash steps of dissociated cells. (M‒O) Debris removal steps for cortex ≥ P7 or midbrain ≥ P0.

***Note:*** When multiple mice are pooled in one reaction, add tissue to Papain-DNase solution one mouse at a time and keep on ice (Figures 4A‒4E).

22. Transfer isolated tissue to prepared Papain-DNase solution in a 50 mL bioreactor tube (Figures 4A–4E).

**CRITICAL:** Ventilated cap bioreactor tubes are used during the whole procedure in order to allow oxygenation of cells critical for cell survival.

23. Independent of age, using a P1000 pipette and filtered pipette tips, pipette up and down 10 times during an initial mechanical dissociation to break down tissue to aid digestion.

**CRITICAL:** Avoid creating air bubbles (Figures 4F and 4G). We prefer mechanical dissociation with a pipette over using a scalpel to keep the number of ruptured cells as small as possible.

24. Incubate tissue in Papain-DNase solution at 37°C for 30 min covered from light in a shaking water bath set to 150 rpm.
25. For brains ≥ P7, briefly interrupt incubation after the first 15 min with manual dissociation described in step 23 (Figure 4H).
26. During the incubation time, prepare the respective buffer or media for sorting and the appropriate collection tubes (0.2 mL PCR tubes) or 1.5 mL microfuge tubes.
27. After 30 min, remove the bioreactor tube containing the dissociated tissue from the water bath and add Solution 2.
28. Mix thoroughly by pipetting up and down to dissolve remaining tissue parts (Figure 4I) (see Troubleshooting Problem 5).
29. Centrifuge at 200 × *g* for 10 min at 20°C–22°C. A cell pellet should be visible (Figure 4J).
30. Remove supernatant and resuspend the pellet in Solution 2.
31. Add 4 mL DMEM/F12 as a wash buffer and mix well.
32. Centrifuge at 350 × *g* for 10 min at 20°C–22°C. A washed cell pellet (smaller than the previous one) should be visible (Figure 4K).
33. For cortices ≥ P7 or midbrain ≥ P0, perform debris removal steps starting from step 34. For younger brains, skip to step 42 (Figure 4L) (see Troubleshooting Problem 6).
34. Remove supernatant and resuspend pellet in 1.5 mL cold PBS per pellet and transfer to individual 15 mL falcon tube per reaction. Perform the rest of debris removal steps on ice.
35. Add 1 mL cold Debris Removal Solution per tube and mix well.
36. Carefully overlay 2 × 1 mL cold PBS per tube using a P1000 pipette with filtered pipette tips.

***Note:*** Prevent mixing by tilting the falcon tubes at a 45° angle while adding PBS. A clear PBS upper layer should be visible over a cloudy cell suspension bottom layer (Figure 4M).

37. Centrifuge at 3,000 × *g* for 10 min at 4°C (with maximum acceleration and brake speed).
38. After centrifugation, an interface containing debris will be visible between layers 1 and 2.
39. Carefully aspirate the top layer and then the debris interface with a P1000 pipette and discard, leaving most of the bottom layer containing the cell pellet undisturbed (Figure 4N).
40. Fill each tube up to 5 mL with cold PBS and gently invert flacon tubes 3 times.

**CRITICAL:** Do not vortex.

41. Centrifuge at 1,000 × *g* for 10 min at 4°C (with maximum acceleration and brake speed). A final pellet will be visible (Figure 4O).
42. Remove supernatant and resuspend pellet in 100–300 μL media depending on the size of the final cell pellet.

**Figure 5:**
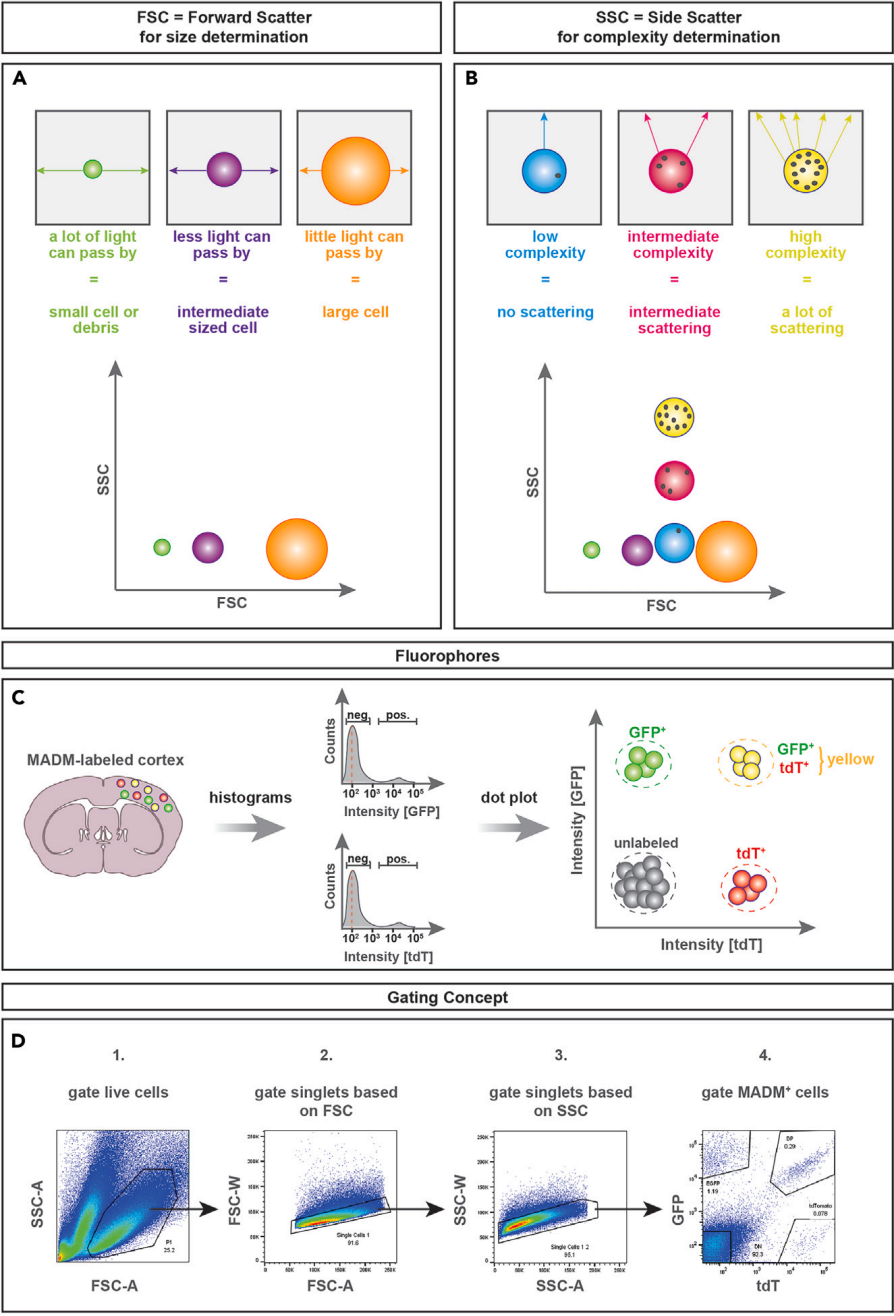
Principle of FACS parameters in order to detect MADM-labeled cells from dissociated brains (A) Size determination based on FSC (forward scatter) parameter. (B) Cell complexity determination based on SSC (side scatter – BD Aria) or BSC (back scatter – SONY SH800) parameter. (C) Display of fluorophore intensity detection for GFP and tdT/mCherry. (D) Dot plots showing the gating concept for MADM-labeled cells from P0 *MADM-19*^*GT/TG*^;*Nestin*-Cre cortex. Use this strategy as a template for your experiment: first set a gate on live cells (gate P1) and then use area (A) and width (W) measurements of forward scatter (gate P2) and side scatter (gate P3) to gate on true singlets. For the final gating step, display fluorescence intensities for GFP and tdT/mCherry from live singlets and place gates on GFP^+^, tdT^+^, and GFP^+^ tdT^+^ cell populations. Abbreviations: FSC-A – forward scatter area; SSC-A – side scatter area; FSC-W – forward scatter width; SSC-W – side scatter width.

***Note:*** Cell-suspension should be milky without visible pieces of tissue.

**CRITICAL:** Cell suspension that is too dilute results in unnecessary extra time during FACS which, depending on the sorting buffer, might lead to low survival rate of cells. Cell suspension that is too concentrated can be further diluted using media at a later stage.

43. Keep single-cell suspension on ice until sorting.

### Sorting MADM-labeled cells for downstream molecular analysis (collection of low cell numbers)

**Timing: 45 min to 3 h**

In this section of the protocol we describe how MADM^+^ cells can be sorted according to their fluorescent colors and collected for downstream molecular analyses such as RNA-sequencing on two different sorting devices: BD Aria III and SONY SH800. We will describe the general protocol for sorting and highlight SONY- or Aria-specific steps.

***Note:*** Successful sorting requires recording of various parameters at the flow cytometer that a) ensure visualization of size and cellular granularity to perform size exclusion (Figures 5A and 5B): FSC (forward scatter), SSC (side scatter) or BSC (back scatter), and b) allow visualization of fluorescence intensity of the MADM labeling in order to properly gate on MADM-labeled cell populations (Figure 5C): GFP and tdT.

***Note:*** Be aware that you need to define the negative and positive signal of your selected fluorophores individually. This is done best by using a histogram to visualize fluorescence intensity. We usually define negative populations by adjusting the voltage of the respective fluorophore to a value that places the maximum of the negative population peak at 10^2^ (Figure 5C). For this purpose, negative populations are cells without MADM-labeling which should be the majority and result in a large peak. The MADM-labeled positive populations will make up a distinct and much smaller peak.

***Note:*** A summary of the complete gating concept for MADM-labeled cells is provided in Figure 5D. First set a gate to discriminate between debris and live cells and thus to select the live cell population. Next, remove doublets by use FSC-A and FSC-W. Then, use SSC-H and SSC-W at Aria or BSC-A and BSC-W at SONY to perform a second round of doublet exclusion. Finally, display GFP and tdT to set your gates for sorting MADM^+^ cells (GFP^+^, tdT^+^ and optionally GFP^+^ TdT^+^ double positive) cells (Figure 5D).

**Figure 6:**
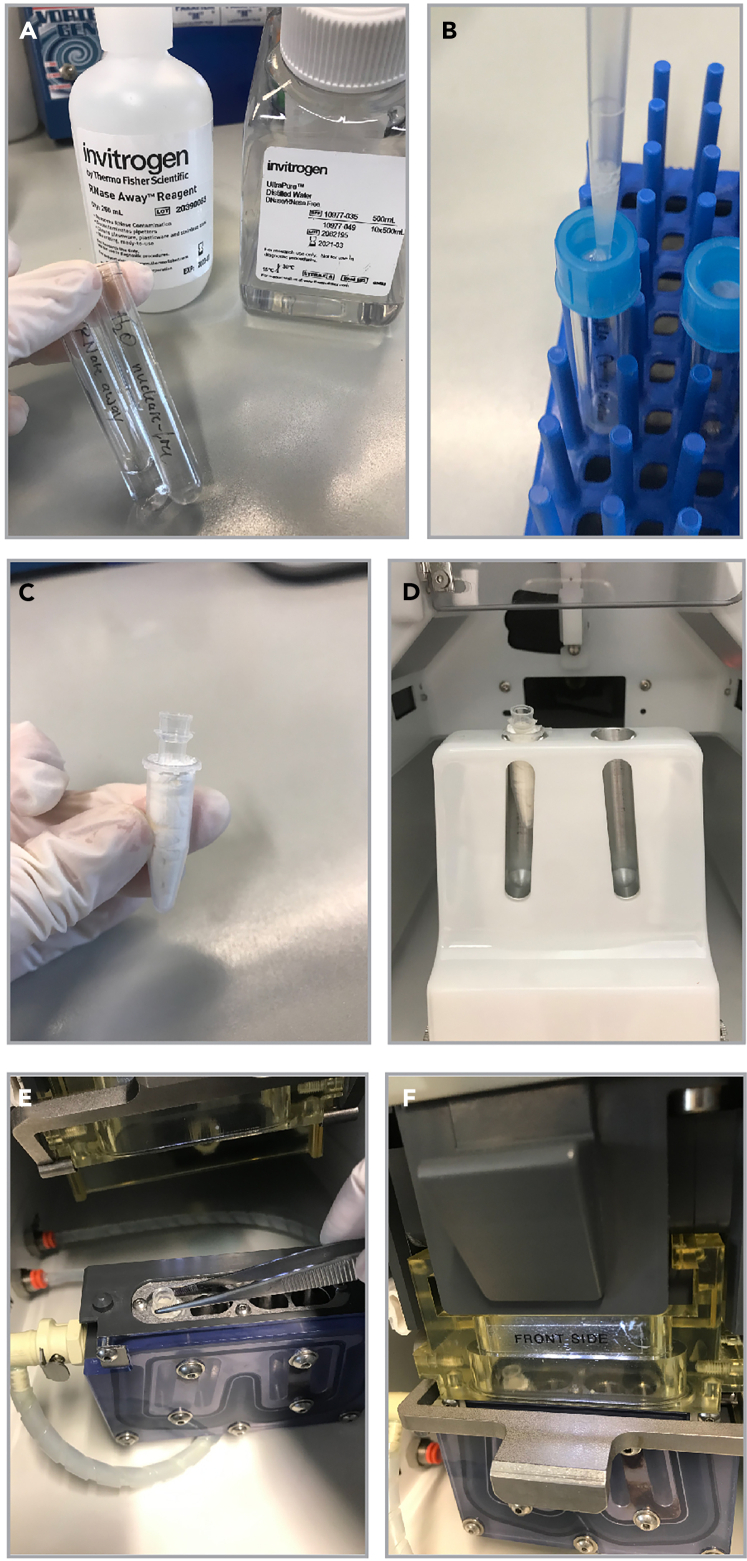
Sorter preparations (A) Preparation of one FACS tube with RNase Away and one FACS tube with RNase-free water for initial washing steps of the flow cytometer. (B) Filtering of the single-cell suspension through a FACS tube with a 40 μm cell strainer prior to sorting. (C) 1.5 mL microfuge tube filled with paper tissue, serving as a holder for 0.2 mL PCR collection tube. (D) Collection tube inserted in 2 way tube holder at SONY SH800. (E and F) Collection tube inserted in 4-way 1.5 mL tube holder at BD Aria.
46. Flush the system with each solution for approximately 1 min. First use RNase Away, then RNase-free water.
47. Filter your entire sample through a 40 μm cell strainer using a P1000 with filter tips (Figure 6B).
48. Dilute your sample with 200–300 μL media, which is additionally pipetted through the cell strainer.
49. Insert test sort collection tubes (empty 1.5 mL microfuge tubes or empty 0.2 mL PCR tubes, depending on your downstream application).

***Note:*** We have chosen to depict the tube holder for 0.2 mL PCR tubes in Figure 6C) into the collection tube holder at SONY (Figure 6D) or BD Aria (Figures 6E and 6F).

***Note:*** Only at BD Aria: Use “test sort” to adjust the side streams in order to allow the side streams to hit the middle of the collection tubes. Afterwards, discard the test sort collection tubes. At SONY, side streams cannot be adjusted by the operator.

**Figure 7:**
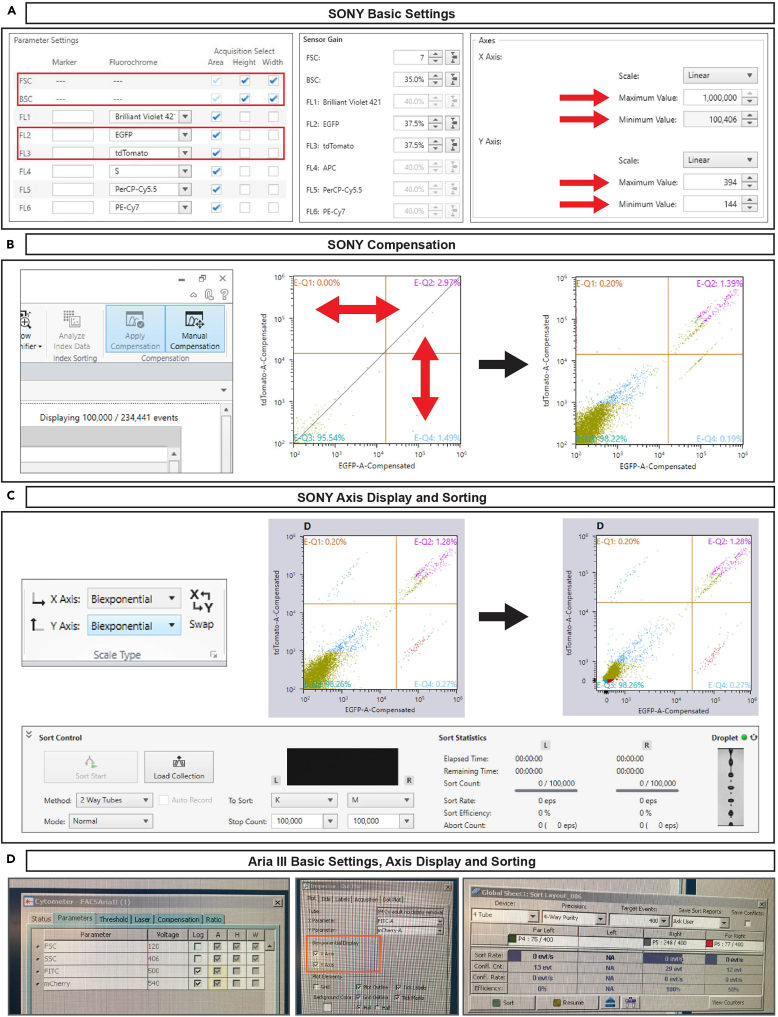
Software settings at the flow cytometer (A) Selection of basic settings (selecting parameters as well as setting detection and threshold settings, left and middle) and adjustment of axis scaling settings (right) at SONY SH800. (B) Performance of manual compensation at SONY SH800 in order to display the GFP^+^ and tdT^+^ populations without spectral overlap within the dot plots. (C) Adjustment of axis display for dot plots by changing logarithmic scale to biexponential display. Note that settings for sorting are instantly visible at the bottom of SONY sorting software. (D) Selection of basic settings (selecting parameters as well as setting voltages, left), adjustment of axis display for dot plots by selecting biexponential display (middle) and setup of sort layout (right) at BD Aria. Note that the sort layout window needs to be attentively opened by clicking on the tab “Sort” and selecting “Sort layout” in BD DIVA software.
51. Select FSC-A, FSC-H, FSC-W, SSC-A, SSC-H, SSC-W, FITC, and mCherry when operating the BD Aria (Figure 7D, left).
52. Sensor gain should be optimized according to signal strength (Figure 7A, middle). For detailed video guide, see Methods video S1.
53. Load your sample and record 30,000–50,000 events in order to set up the gating parameters with the recorded cells.

***Note:*** The baseline settings at SONY might not show all FSC and BSC events within the range of the axes. You can manually adjust the axes ranges by right-clicking on the respective axis, selecting “properties” and then entering a different minimum or maximum value for the axis scale (Figure 7A, right).

***Note:*** At BD Aria, detectors for FITC and mCherry will not show a spill-over of one fluorophore to the other, therefore no compensation is needed. However, the detector configuration of SONY requires compensation. With MADM, we recommend to perform manual compensation at SONY by dragging the respective cell populations with the cursor in the correct direction (Figure 7B).

***Note:*** Dot plots showing fluorescence intensities usually display logarithmic scales by default. We recommend to switch to biexponential display for an inclusive visualization of all acquired cells and thus a better discrimination of negative and positive populations (Figure 7C for SONY; Figure 7D, middle, for BD Aria).

44. Ensure that the cytometer is correctly set up for your sorting experiment.
  a. Use the 100 μm nozzle.
  b. Select/turn on the 488 nm and 561 nm lasers.
  c. Set the temperature for sample and collection tubes to 4°C.
45. Prepare one FACS tube with RNase Away and one FACS tube with RNase-free water (Figure 6A).

50. In the parameters window, select FSC-A, FSC-H, FSC-W, BSC-A, BSC-H, BSC-W, GFP, and tdT when operating the SONY SH800 (Figure 7A, left).

54. Prepare the collection tubes for your sorted cells.
  ***Note:*** The type of tube depends on your downstream analysis:
  a. For low input bulk RNA-seq, prepare 0.2 mL PCR tubes containing 4 μL Lysis buffer as collection tubes for your sorted cells. Since neither sorting device can hold 0.2 mL PCR tubes in its collection tube holders, prepare custom-made PCR tube holders for sorting. We show this type of collection tubes as examples in the figures of our protocol (Figures 6C–6F):
    i. Take 1.5 mL microfuge tubes and cut off the lid.
    ii. Fill the 1.5 mL tubes with paper tissue or Parafilm (Figure 6C).
    iii. Insert a PCR tube in the middle of the stuffed 1.5 mL tube.
    iv. After sorting, PCR tubes can be taken out of the 1.5 mL tubes using forceps and PCR tube holders can be reused by inserting a new PCR tube.
  b. For 10X Genomics scRNA-seq, prepare 1.5 mL microfuge tubes containing 100 μL media.
  c. For proteomics, prepare 1.5 mL microfuge tubes containing 50 μL lysis buffer (LYSE-NHS from PreOmics iST-NHS kit).
55. For sorting at SONY: the sorting options are already visible at the bottom of the software wizard (Figure 7C, bottom), thus you can instantly select the correct tube holder (in our case: 2 way tubes).
56. For sorting at Aria: open the “sort layout” in BD DIVA software, choose 4 tube collection device and select 4-way purity as the sorting mode to sort your sample (Figure 7D, right).
57. Assign the sorting gates to the collection tubes.
58. Start sorting.

***Note:*** Depending on your experimental needs and the MADM lines used, either set a maximum number of labeled cells that shall be collected or collect an indefinite number of cells to obtain as many cells as possible.

59. Note down the number of sorted cells from each selected cell population when sorting at BD Aria.

***Note:*** As opposed to SONY sorting software, BD DIVA software does not save the number of sorted cells from each sample.

***Note:*** For bulk RNA-seq: The RNA Lysis buffer described in this protocol was used and optimized for single cells and low amounts of cells (max. 400 cells). If a greater number of cells will be collected with FACS, a different lysis buffer needs to be used. For details, please see.^11^

***Note:*** For bulk RNA-seq: Design a layout for collecting your samples. Keep in mind that you may want to sort a higher number of cells (up to 400 cells) as a positive control for your sequencing experiment in one of the wells. For this purpose, you can make use of the MADM-unlabeled cells as they are not limited in cell number.

**CRITICAL:** Prior to sorting, consult with your sequencing facility regarding the plate layout since empty wells and a positive control might be needed at specific positions.

60. Once sorting is completed, proceed with your collection tubes to the next steps depending on the further downstream application (see Troubleshooting Problem 7).

***Note:*** For bulk RNA-seq: transfer the entire volume of the collected sample into an empty well in a 96-well plate, which should remain on dry ice throughout the procedure. Be fast with pipetting since samples freeze in the pipette tip if this step is executed too slowly.

Methods video S1. Video guide to software settings at SONY and BD Aria flow cytometers, related to step 52

Collect all replicates for your RNA-sequencing experiment in the same 96-well plate. Either fill the entire plate on a single day, or add individual samples on a day-to-day basis. When adding few samples over a time course of several days or weeks, store the plate at ‒80°C until further processing or additional samples can be added.

***Note:*** The described setup with collecting samples on a 96-well plate allows sample collection for RNA-sequencing on consecutive days. However, sample collection and handling may need to be adapted to individual use and respective purposes.

After sorting of all required samples is completed, seal the plate with AluminaSeal, freeze immediately on dry ice and store at ‒80°C until further processing. In order to ensure maximum sequencing efficiency plates should be processed within three months after collection.

***Note:*** For 10X genomics scRNA-seq: spin down the tubes with your sorted cells, remove the supernatant and resuspend the pellet in Chromium Next GEM Master Mix. Proceed with sample preparation according to the 10X genomics protocol used by your sequencing facility.

***Note:*** For proteomics: Immediately after sorting, boil your samples for 10 min and then continue with the protocol of the iST-NHS kit (PreOmics).^7^

**CRITICAL:** Since only one sample can be sorted at any time, it is important to determine the required number of cells for your sequencing experiment beforehand. The longer a sample is kept on ice before it is processed by FACS, the higher the amount of dead cells. Instead of preparing single-cell suspensions from multiple samples at the same time, consider to prepare single-cell suspensions in multiple batches to keep the waiting time of individual samples on ice short and thus increase the cell survival rate.

## Expected outcomes

Mechanical and enzymatic tissue dissociation required to obtain single-cell suspensions usually results in a single-cell suspension that contains both live cells and debris. The flow cytometer will detect debris and live cells as individual events. Each event will be displayed as one dot. In order to ensure correct sorting of your desired cell populations, it is crucial to remove as much debris as possible from your cell suspension and your FACS plots. In order to remove debris from FACS plots, a selective gating strategy that excludes debris and doublets will greatly advance the rigor of your sorting experiment. The gating concept shown in Figure 5D provides a template for the gating strategy that first gates on live cells (gate P1) and then uses area (A) and width (W) measurements of forward scatter (gate P2) and side scatter (gate P3) to gate on true singlets. For the final gating step, display fluorescence intensities for GFP and tdT from live singlets and place gates on GFP^+^, tdT^+^, and GFP^+^ tdT^+^ cell populations.

**Figure 8:**
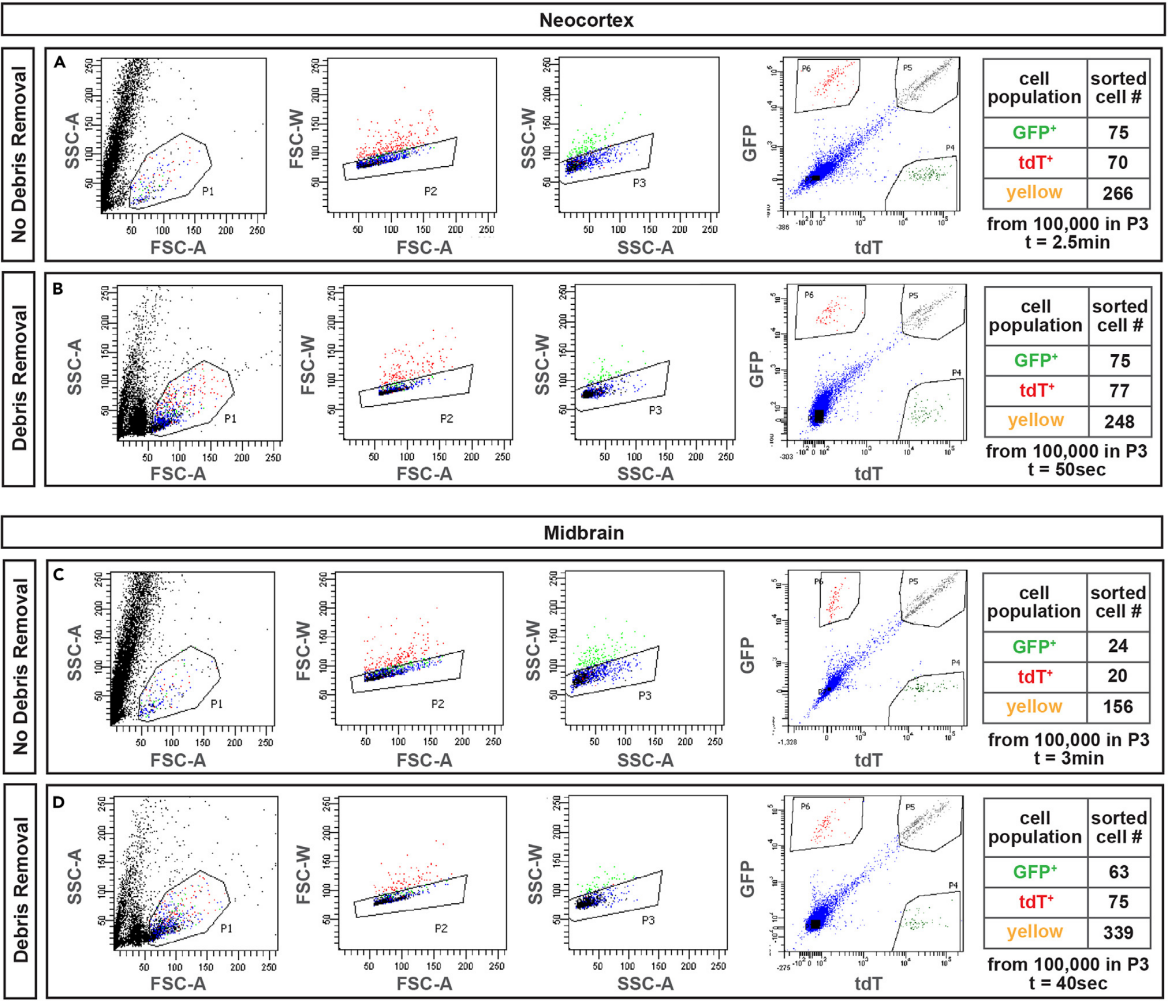
Representative FACS plots of adult MADM-labeled cortices and midbrain (A‒D) FACS plots of single-cell suspension prepared from 2-month old *MADM-19*^*GT/TG*^;*Nestin*-Cre mice. Dot plots are shown for neocortex (A and B) and midbrain (C and D). For each brain region, a comparison between before (A and C) and after (B and D) debris removal steps is illustrated. For each panel, the left most plots show live cells (outlined gate P1) versus debris (black dots). An enrichment in live cells by debris removal steps can be observed in both cases. The second and third plots show the gating of singlets by forward scatter (P2) and then by side scatter (P3). The fourth plots show the separation of fluorescently labeled cells in green/GFP^+^ (P4), red/tdT^+^ (P6) and yellow/GFP^+^tdT^+^ (P5). The numbers of sorted cells and sorting duration are listed in the tables on the far right and show that debris removal steps result in faster sorting in both regions and a higher yield particularly in white-matter rich midbrain.

With increasing age of your mice, the proportion of debris in the single-cell suspension increases drastically. Debris is mostly derived from myelin particles and dying cells. Thus, the higher the myelin content in your brain region of interest, the higher the amount of debris. We thus recommend debris removal in order to increase the relative proportion of live singlets in your cell suspension. This strategy allows faster sorting of MADM-labeled cells (compare Figures 8A and 8B as well as Figures 8C and 8D). In the adult neocortex and midbrain, one round of debris removal results in a 3- or 4.5-fold increase in recording speed, respectively (cortex without debris removal: recording 100,000 cells in 150 sec, cortex with debris removal: recording 100,000 cells in 50 sec; midbrain without debris removal: recording 100,000 cells in 180 sec, midbrain with debris removal: recording 100,000 cells in 40 sec). Interestingly, debris removal also resulted in a 3-fold increase of sorted cell yield from the midbrain, while the number of sorted cells from the cortex was not affected by debris removal (compare tables in Figures 8A and 8B as well as Figures 8C and 8D).

## Quantification and statistical analysis

Purified MADM-labeled cell populations can be used for various downstream molecular analyses such as RNA-seq, DNA-seq or proteomics. In the above protocol, we have described the sorting process for low input bulk RNA-seq, 10X Genomics scRNA-seq and proteomics.^5^^,^^6^^,^^7^^,^^3^^,^^15^

## Limitations

Our protocol for preparing single-cell suspensions has been optimized for embryonic time points and postnatal mice up to 2 months of age. Application of the protocol for later adult stages might require additional optimization due to increased cell death and increased myelin-containing debris.

MADM is a technology that can be used to sparsely label cells with fluorescent markers.^1^ We have developed a genome-wide library of MADM mice to employ MADM to study the vast majority, nearly genome-wide (>96%), of autosomal genes in the mouse genome. Distinct MADM lines exhibit different recombination frequencies and thus the amount of cortices to be pooled and the number of cells to be obtained strongly depends on the respective MADM line chosen. However, collection of up to 400 cells is possible for many MADM lines, even without pooling multiple cortices.

To provide an estimation of the feasibility of distinct MADM lines for downstream molecular analysis, the following table offers an overview of cell yield from individual P0 cortices of distinct MADM lines using MADM-labeled GFP^+^ cells of the *Emx1* lineage as an example.

**Table:** 

MADM line	MADM recombination frequency^1^	Time point	# Of GFP^+^ cells
MADM-17	intermediate	P0	350–400
MADM-7	dense	P0	1,000
MADM-19	dense	P0	∼10,000
MADM-11	dense	P0	∼12,000–15,000

Note that cell yield not only depends on the MADM line of choice, but also on the age of the mice. For instance, we pooled cortices of 3 mice of the MADM-7 line in order to obtain 30‒50 GFP^+^ cells of the *Emx1* lineage at E12.5.

Consequently, MADM is most suitable for downstream applications that require limited biological material, up to a few thousand cells and/or for particular single-cell applications. In our experience, single-cell RNA-seq or very low input bulk RNA-seq using the SMARTer method has always provided us with robust results.^5^^,^^11^ Thus, the minimum required number of sorted cells is as small as 1. However, the number of individual mice needed to investigate a specific cell population or biological process strongly depends on your scientific question.

## Troubleshooting

### Problem 1

Low number of experimental mice (step 1).

### Potential solution 1

Make sure the genetic background of your mice does not exhibit negative effects on their mating performance. Increase the number of breeding cages to gain a good number of F1 or F2 mice to start with. Check whether you have correctly followed the breeding strategies published.^12^ Wrongly genotyped or selected parental mice can result in strongly decreased efficiency in the generation of experimental animals.

### Problem 2

Low breeding performance (step 1).

### Potential solution 2

Discuss with your veterinarian how to optimize the mating conditions (e.g., by providing special food or housing conditions).

### Problem 3

Highly variable results in MADM lines with low recombination frequency (step 1).

### Potential solution 3

Analysis of all 19 MADM lines showed that different MADM chromosomes harbor distinct recombination frequencies [sparse, intermediate or dense^1^]. The required number of experimental animals and the number of samples used for analysis need to be scaled up when working with a MADM chromosome that gives sparse labeling. We recommend pooling multiple cortices from several animals to avoid introduction of an analysis bias when only using one animal from a low recombination line.

### Problem 4

Extracted brain contains high amount of undesirable red blood cells (step 8).

### Potential solution 4

Brain vasculatures become more mature as the brain age. When dissected brain looks visibly pink or is covered with blood vessels, red blood cells will become dissociated with the tissue and can be found in the pellet. Although red blood cells can usually be separated by gating at the sorter, too many red blood cells can make gating of desired cells difficult and also increase the time of sorting. Depending on the brain region of interest, we recommend performing HBSS perfusion for mice P7 or older prior to extraction of the brains.

### Problem 5

Visible pieces of tissue remain undissolved after 30 min enzymatic digestion and mechanical dissociation (step 28).

### Potential solution 5

Several solutions may improve tissue dissociation. 1) Use less tissue per reaction to optimize enzyme to tissue ratio. 2) Introduce additional brief mechanical dissociation intervals during enzymatic digestion. 3) Increase duration of enzymatic digestion. Cell-suspension should be milky without visible pieces of tissue at the end of the procedures.

### Problem 6

Difficult to gate live cells due to large amount of debris (step 33).

### Potential solution 6

Repeat using debris removal step in your protocol. This is important in particular for older brains and white matter-rich brain regions. After debris removal, live cells should be more isolated and visible and therefore easier to gate.

### Problem 7

Significant cell death after sorting (step 60).

### Potential solution 7

Sorted cells may be subjected to conditions that are not favorable for survival. This is relevant for single-cell RNA-seq or other downstream procedures where cells are not immediately lysed after sorting. Several considerations should be taken into account: 1) Temperature: both sorting temperature and sorted cells should be kept at 4°C to minimize cell death. 2) Sorting solution: depending on the downstream analysis of choice, consider sorting cells into serum containing medium to improve survival. 3) Time of sorting: the time of sorting the desired number of cells can be shortened by increasing the sorting rate within limits of the sorter or by removing debris in your samples by adding debris removal steps.

## Resource availability

### Lead contact

Further information and requests for resources and reagents should be directed to and will be fulfilled by the lead contact, Simon Hippenmeyer (simon.hippenmeyer@ist.ac.at).

**Technical contact**

Additional requests regarding technical details should be directed to the technical contacts, Nicole Amberg (nicole.amberg@meduniwien.ac.at) and Giselle Cheung (giselle.cheung@ist.ac.at).

### Materials availability

This study did not generate new unique reagents.

### Data and code availability

No custom code or dataset was generated in this protocol.
